# Virtual Biopsy in Soft Tissue Sarcoma. How Close Are We?

**DOI:** 10.3389/fonc.2022.892620

**Published:** 2022-07-01

**Authors:** Amani Arthur, Edward W. Johnston, Jessica M. Winfield, Matthew D. Blackledge, Robin L. Jones, Paul H. Huang, Christina Messiou

**Affiliations:** ^1^ Division of Radiotherapy and Imaging, The Institute of Cancer Research, Sutton, United Kingdom; ^2^ Sarcoma Unit, The Royal Marsden National Health Service (NHS) Foundation Trust, London, United Kingdom; ^3^ Division of Clinical Studies, The Institute of Cancer Research, London, United Kingdom; ^4^ Division of Molecular Pathology, The Institute of Cancer Research, Sutton, United Kingdom

**Keywords:** sarcoma, MRI, radiomics, virtual biopsy, quantitative MRI (qMRI), radiology pathology correlation, soft tissue sarcoma (STS), imaging biomarker

## Abstract

A shift in radiology to a data-driven specialty has been unlocked by synergistic developments in imaging biomarkers (IB) and computational science. This is advancing the capability to deliver “virtual biopsies” within oncology. The ability to non-invasively probe tumour biology both spatially and temporally would fulfil the potential of imaging to inform management of complex tumours; improving diagnostic accuracy, providing new insights into inter- and intra-tumoral heterogeneity and individualised treatment planning and monitoring. Soft tissue sarcomas (STS) are rare tumours of mesenchymal origin with over 150 histological subtypes and notorious heterogeneity. The combination of inter- and intra-tumoural heterogeneity and the rarity of the disease remain major barriers to effective treatments. We provide an overview of the process of successful IB development, the key imaging and computational advancements in STS including quantitative magnetic resonance imaging, radiomics and artificial intelligence, and the studies to date that have explored the potential biological surrogates to imaging metrics. We discuss the promising future directions of IBs in STS and illustrate how the routine clinical implementation of a virtual biopsy has the potential to revolutionise the management of this group of complex cancers and improve clinical outcomes.

## Introduction

The paradigm shift in radiology from an imaging-based to a data-driven specialty has been unlocked by synergistic developments in imaging biomarkers (IB) and computational science including artificial intelligence (AI) (1). This is propelling imaging to the forefront of oncology and advancing the capability to deliver “virtual biopsies” (2). Virtual biopsies are defined in this review as an imaging method, or collection of imaging methods, which provide similar information about the structure, function and pathology of a tissue to that obtained from histopathology. The ability to non-invasively probe tumour biology both spatially and temporally would fulfil the potential of imaging to inform management of complex tumours; improving diagnostic accuracy, providing new insights into inter- and intra-tumoral heterogeneity and individualised treatment planning and monitoring (3).

Neuroimaging has long served as the test bed for imaging innovation, afforded by the size and immobility of the brain, which allows for repeated acquisition of high quality images. These characteristics are shared with soft tissue sarcomas (STS). STS are often large masses located in the extremities and imaging is therefore not prone to motion and respiratory artefacts encountered in thoraco-abdominal imaging. Further, image-guided biopsy of STS of the peripheries is usually straightforward with low morbidity and can therefore serve as biological validation for IB studies. The primary treatment for local disease is surgery which also provides opportunities for radiology-pathology correlation, which is a crucial component of IB development as non-invasive surrogates for tumour biology (4, 5). Leveraging on these features, we should be recruiting the best innovators and technology to STS research. These tumours provide an ideal testing ground for translational research which can then be transferred to other cancer types but also present a significant clinical need for better diagnostics and treatments. STS are rare tumours of mesenchymal origin that account for 1% of all adult cancers with over 150 histological subtypes and notorious heterogeneity, both between patients and within a tumour itself (6). The combination of inter- and intra-tumoural heterogeneity and the rarity of the disease remain major barriers to effective treatments (7–10). Whilst initial local control is often achieved, distant recurrence is frequent and associated with a generally poor prognosis, with a median survival reported between 12 and 20 months (11–17). Without methods to tackle the vast heterogeneity, we fail to confidently stratify patient risk and identify patients most likely to benefit from treatment and in turn, continue to apply a “one size fits all” approach to therapy. As we advance our biological understanding of these sarcomas, combining this with innovative imaging tools and reliable IBs could dramatically alter the landscape of such a devastating disease.

Herein, we provide an overview of the long-standing challenges facing our ambition to advance treatment and improve clinical outcome for STS patients. We describe the advances in IBs and computational imaging in STS that can help overcome these challenges and the realising promise of “virtual biopsies”. We focus on the studies that have sought to correlate imaging with biology, the development of quantitative MRI parameters in STS and radiomics that has been applied to this group of cancers. We then discuss the challenges and future work that is required to discover, validate and translate IBs into the clinical setting for these patients.

## Imaging Biomarkers in STS

With the rapid growth of IBs and computational image analysis capabilties in the past decade, radiological images are now increasingly recognised as valuable datasets that can provide complementary information to aid the diagnosis and management of patients with STS.

Improved imaging tools are required for STS tumours as is a detailed understanding of their relationship with histological changes which is vital for IB development. An IB is defined by the National Institutes of Health (NIH) as “characteristics that are objectively measured and evaluated as an indicator of normal biological processes, pathogenic process or biological responses to therapeutic intervention” (18, 19). IBs can be numerical (quantitative) or categorical (quantitative value or qualitative). An IB roadmap has been produced to accelerate the translation of IBs from conception into useful clinical tools (5). This includes two translational gaps that must be crossed. The first is to become medical research tools if the IB is confirmed to reliably test medical hypotheses, and the second as clinical decision-making tools if the IB is confirmed to be clinically useful and has been clinically validated (20). In order to bridge these translational gaps, IBs need to pass through a series of domains: discovery (Domain 1), validation (Domain 2) and qualification with ongoing technical validation (Domain 3), including technical validation (e.g. repeatability, reproducibility and availability) and biological and clinical validation (e.g. relationship with disease state, diagnostic, prognostic and predictive capabilities) (5).

In STS, IBs have the potential to provide non-invasive insights into tumour biology over time without sampling bias, including in relation to intra–tumoural heterogeneity (21, 22). Furthermore, they bypass any technical and clinical difficulties of biopsy for diagnosis and treatment monitoring, allowing improved patient risk stratification and treatment planning. The capabilities of multiparametric magnetic resonance imaging (MRI) present an attractive tool for quantitative IB development for clinical adoption (23). The ability to capture multiple parameters in a single scan, with the potential to explore multiple biological properties which contribute to intratumoural heterogeneity is highly appealing (3).

### Quantitative MRI

**Figure 1:**
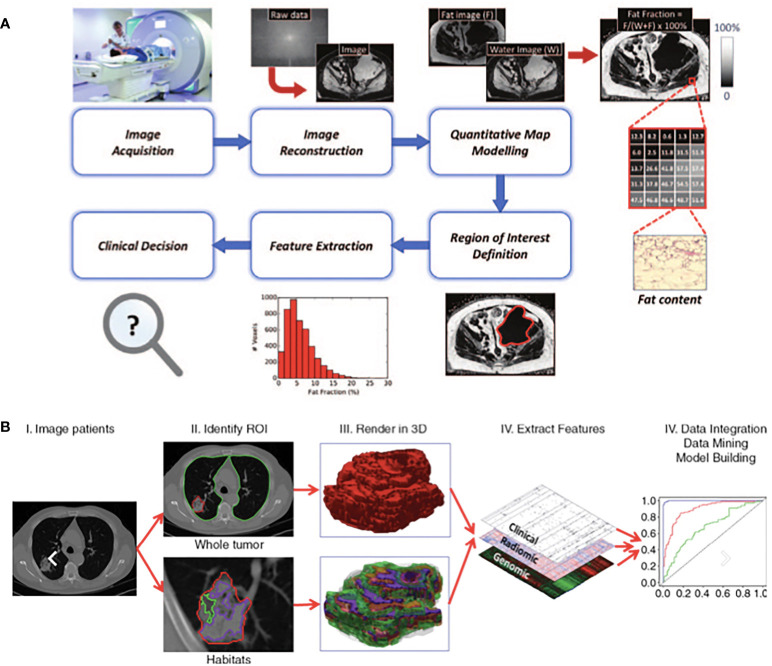
Exemplars of typical quantitative imaging in **(A)** and radiomic workflows in **(B)** taken from Blackledge et al. (22) and Gillies et al. (24) respectively. **(A)** Quantitative imaging Involves a detailed process of steps. Following image acquisition and image reconstruction, quantitative maps are developed either by the scanner or offline and these maps differ from the parent images as each voxel has an unit of measurement. Multiple regions of interest are often selected, features extracted accordingly to aid clinical decisions. **(B)** Radiomics involves a multi-step process. Following acquisition of high quality images, regions of interest and/or habitats are defined. These are reconstructed into 3D. Radiomic features are extracted and models are developed with correlation of these extracted features and pre-defined clinical outcomes of interest.

Quantitative MRI is a multi-step process that requires image acquisition, reconstruction, segmentation and often mathematical modelling of parameters prior to feature extraction (**Figure 1**) (2). A semi-quantitative MRI parameter already in use in clinical practice is contrast-enhanced (CE-)MRI, where the difference in signal after intravenous injection of a gadolinium-based contrast agent is used as a surrogate for tumour vascular perfusion (22). Examples of fully quantitative parameters are Dixon MRI which enables fat content in tumours to be visualised and quantified and apparent diffusion coefficient (ADC) measurements from diffusion-weighted (DW)-MRI which maps tissue cellularity (22, 25–27). ADC is rapidly growing in use in oncology because of the widespread availability of DW-MRI on clinical scanners, ease of acquisition (not requiring additional equipment or injection of contrast agents), excellent repeatability and large number of biological and clinical validation studies (22, 27, 28). These factors make it an attractive IB, and as such, ADC has emerged as a useful quantitative IB in STS which has led to its incorporation in the European Organisation of Research and Treatment of Cancer (EORTC) guidance on standard of care MRI in STS (29–31).

### Radiomics

The emerging field of “radiomics” may also serve to identify IBs for use in STS. Radiomics describes the extraction of features from radiological images, converting them to mineable high-dimensional data and searching for correlations with defined clinical variables. It relies on the extraction of both semantic (radiology lexicon such as size and shape) and agnostic (quantitative descriptors such as texture and histogram) features, interrogating tumour morphology and behaviour on a deeper scale (32, 33). Correlative assessment of these features with clinical variables may drive generation of diagnostic, prognostic or predictive models for variables of interest, to act as complimentary non-invasive decision support tools (**Figure 1**) (24). Radiomics may identify an individual feature or set of features (a signature) that could be utilised as an IB. Radiomics is particularly attractive as it can explore and discover clinically relevant IBs without the need for new or complex imaging systems.

A typical radiomics workflow consists of multiple steps: high quality image acquisition, preprocessing of the images to ensure uniformity of scans, tumour segmentation and feature extraction. Extracted features are combined with other mineable data and correlations for clinical outcomes of interest are explored (24). Cancer types where radiomics has shown promise include lung cancer, glioblastoma and prostate cancer where radiomics has been used to distinguish benign from malignant tissue, measure the aggressiveness of tumours, aid diagnosis and selection of biopsy sites (24, 34–39). In addition, radiomics has identified imaging phenotypes suggestive of patient prognosis and predictions of treatment response (24, 34, 36–42). The use of radiomics in a highly heterogeneous malignancy like STS could deepen our understanding of the biology across the entire tumour volume, and its correlation with clinical outcomes. To date, preliminary radiomic studies in STS have focused on correlation of radiomic features with pathological grading and prognosis (43–47). Multiple studies have demonstrated an association of final histopathological grade in the surgical specimen on tissue examination with extractable radiomic features, including the ability to discriminate between tumours of low and high grade (43, 44, 47). In addition, radiomic features were predictive of distant metastases, early response to neoadjuvant chemotherapy and clinical outcome (46–50). The studies performed in STS remain limited to single centre cohorts with small patient numbers and imaging performed on single scanners. Radiomics requires large datasets to allow confident training of models and for robust conclusions to be drawn, and therefore, the rarity of STS and resultant small datasets has significantly hindered the progress of applying radiomics to “real life” clinical practice. Solutions for dealing with smaller datasets are emerging, such as using internal cross-validation to test radiomic models removing a dependence on large independent datasets (51). Overcoming this challenge would pave a bright future for radiomics in STS and its potential to supply unparalleled information about tumour heterogeneity and its impact on tumour behaviour and clinical outcome.

### Unlocking the Potential of IBs With Computational Imaging

Quantitative MRI and radiomics require time-consuming processing and analysis and produce a vast amount of complex data which make interpretation challenging and prohibit translation as clinical decision making tools. Recently, AI is gaining traction within oncology as offering a complementary suite of technologies to drive uptake of IBs in clinical practice. Defined as the “development of computer systems able to perform tasks normally requiring human intelligence”, AI offers a solution to the time-consuming and complex image processing analyses required for some MRI parameters and for radiomics. It encompasses machine learning and its subset deep learning, each with multiple algorithms, and may prove an indispensable tool for complex data analysis (2, 52, 53). The integral radiomics workflow step of image delineation requires expert radiological input and is time consuming, particularly in the context of multiple tumour lesions or large, complex tumours. Deep learning can train and use algorithms for automated segmentation of tumours without the requirement for human intervention (54). In addition, it can analyse numeric data from predefined radiomic features as well as designing its own radiomics features from the direct analysis of images (55). This holds potential as a attractive tool, maximising the amount of “hidden data” that can be automatically extracted from radiological images, whilst addressing the real life constraints of time consuming segmentation and post processing.

## Diagnosis

The current gold standard for diagnosis in STS remains histopathological examination of biopsy or surgical specimen tissue. Routinely, a pathologist will determine histological subtype, histological grading based on the three-tiered histopathological grading system of the French Federation of Cancer Centers Sarcoma Group (FNCLCC) and therapeutic response demonstated by percentage of remaining viable cells when required. If applicable to the suspected histological subtype, the pathologist will also routinely request ancillary tests to aid diagnosis. This includes immunohistochemistry (for example for proliferation index Ki67, specific proteins and immune cell staining), cytogenetics and molecular analysis for relevant alterations (56, 57). In addition to tumour size, grade by FNCLCC is the most important prognostic factor in STS (58–60). However, whilst core biopsy is the usual modality for obtaining tissue for diagnosis, it is unable to capture the full extent of heterogeneity due to sampling error, and therefore can have the potential to misrepresent histopathological grade (44, 61, 62). In particular, under-grading of STS on core biopsy is recognised (62). Furthermore, given the disease rarity and degree of heterogeneity, accurate diagnosis by non-specialist pathologists can be difficult. In fact, a previous study conducted across 3 European regions confirmed a concordance of only 56% in histopathological tissue examination by primary diagnosis and secondary opinion (63). The main discrepanices were namely histological grade (43%), histological type (24%), subtype (3%) and grade plus subtype or grade plus histological type (29%). A similar finding was reported in a further study which found a 71% concordance in diagnosis between primary and referral centres, with 16.4% major discrepancies and 11.8% minor discrepancies (64).This is complicated by the qualitative nature of histopathological measures used for diagnosis, where inter-observer discrepancies may lead to poor reproducibility and carries the potential to impact patient treatment planning. These findings support the drive to centralise sarcoma care into specialist centres.

Ultimately, we frequently fail to capture the heterogeneity within complex lesions like STS with tissue biopsy alone, and despite the substantial strides made in our biological understanding and management of these diseases, this remains incomplete for most subtypes. Imaging can overcome these challenges either by guiding precision biopsy of higher grade tissue, or by providing biological information or correcting underestimation of grade with direct IBs acting as a surrogate for underlying biological features. Furthermore, efforts to standardise the acquisition of scans will improve accuracy and consistency and could contribute to more robust clinical decision making (65). Below we discuss the evolution of IBs in STS over the past few years.

### Conventional Imaging

The potential of biopsies to misrepresent grade in STS is well established (62). With regards to grading tumours, the primary goal of imaging has remained to achieve the equivalent of *in vivo* microscopy, or provide a complimentary diagnostic aide. In order to overcome the limitations of invasive core biopsies in STS, many MRI studies have sought to test the correlative power of imaging with tumour grade, comparing findings to histopathological examination of specimens based on FNCLCC. In 2008, Liu et al. correlated peripheral tumour growth pattern on MRI with histopathological grade in 59 STS patients, and found poorly defined margins in 60% of high grade tumours, versus well-defined margins in 60% of low grade tumours (66). This has been reinforced in a retrospective study by Zhao et al. where increased intensity of peritumoural contrast enhancement on imaging in higher grade tumours was the strongest independent indicator of histopathologically confirmed high grade STS (67). In addition, a study by Crombe et al. validated findings by Zhao et al. and demonstrated that the three following features of peritumoural enhancement, necrosis and heterogeneity in signal intensity on MRI could allow up-grading of underestimated tumour grade (61). This study also undertook survival analyses and determined that two out of three of these criteria were independent prognosticators of worse metastasis free survival and overall survival through uni- and multi-variable analysis (61).

Computed tomography (CT) sensitivity to detect tissue necrosis has also been utilised to demonstrate improved accuracy of grading in leiomyosarcoma (LMS) when combined with histopathological assessment of tissue, versus histopathology alone (68). Further to tumour grade, studies have explored the CT features of STS that could aid diagnosis, exploiting features such as fat content, margin status and presence of septations. An example of successful utilisation of these imaging parameters is in liposarcoma, a group of STS which can be frequently distinguished radiologically, without the requirement of a diagnostic biopsy (69). Contrast-enhanced CT, which is generally the standard investigation for retroperitoneal sarcomas, can be used for site confirmation and often tissue composition, and can confidently delineate well-differentiated liposarcoma (WDLPS) and angiomyolipoma (70, 71).

### Positron Emission Tomography (PET)/CT

The value of PET/CT in assessing histological grade in STS has been explored. Using ^18^flurodeoxyglucose-positron emission tomography (^18^FDG-PET)/CT, the maximun standardised uptake value (SUVmax) of a lesion was demonstrated to correlate with the FNCLCC. In two meta-analyses, SUVmax was able to distinguish high-grade versus lower-grade STS such that this imaging technique could improve the diagnostic accuracy of core biopsies (72, 73). However, both meta-analyses reported poor methodological quality and lack of comparable parameters across studies, preventing confident conclusions to be drawn.

### Quantitative MRI

Advancements in MRI capabilities has allowed the derivement of quantitative parameters that allow insight into biological features of tumours for example fat, vascularity and cellularity, as well as the spatial heterogeneity across an entire tumour. These parameters may represent translatable IBs that could improve accurate diagnosis. Although the multitude of potential parameters is expanding, some remain in early stages of exploration (**Table 1**). Exploiting MRI features of fat fraction and contrast sequences, liposarcomas can be distinguished from other STS, and provide information about the margin, shape, as well as internal architecture and properties (70, 75, 76). In a group of retroperitoneal sarcoma patients, MRI fat fraction strongly correlated with histopathological fat content on the surgical excision sample (22).

**Table 1:** Summary of the quantitative imaging techniques described in this paper and that represent potential IBs.

MRI technique	Exemplar images	Exemplar parameters	Proposed biological surrogate
Dixon	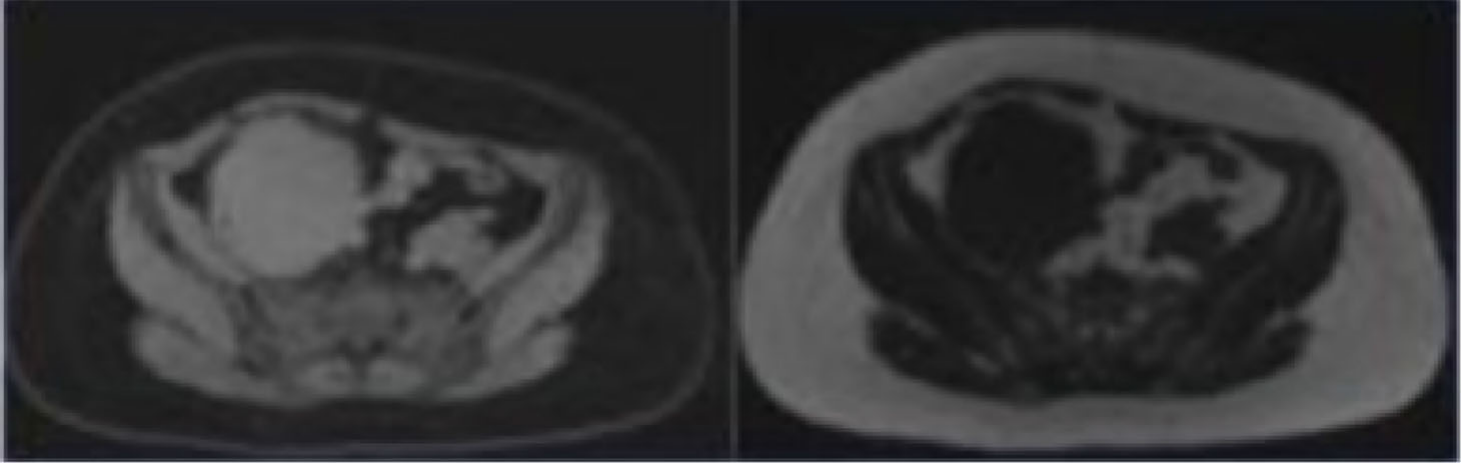	Fat fraction (FF)	Fat content
Diffusion-weighted	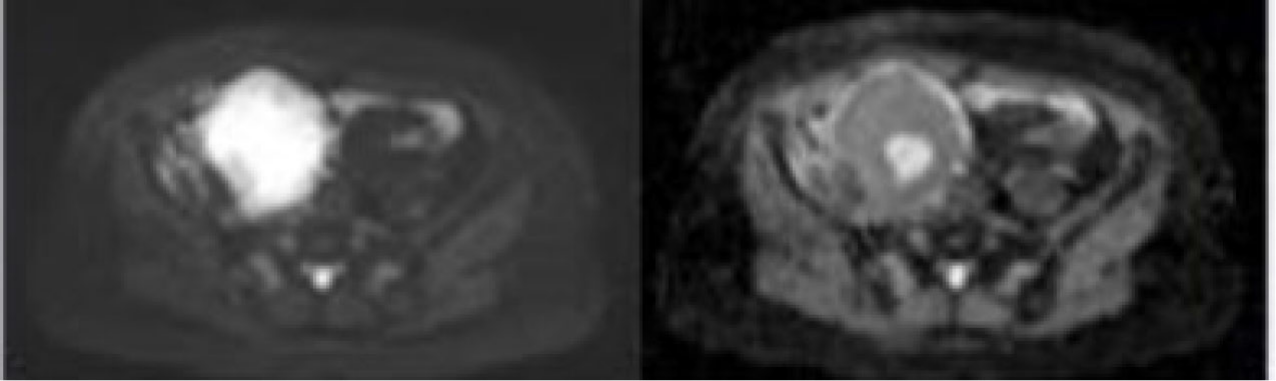	Apparent diffusion coefficient (ADC)	Cellularity
Dynamic contrast-enhanced	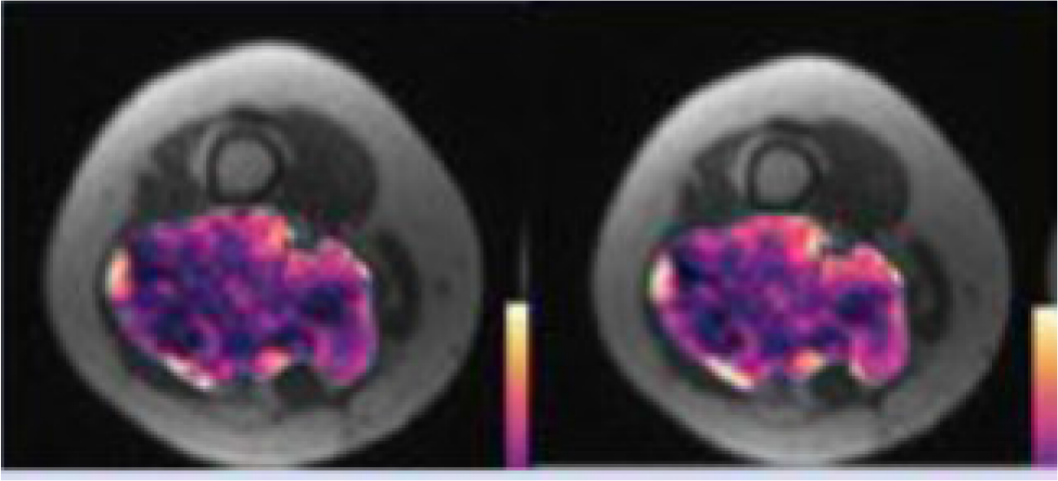	Volume transfer constant (K^trans^) Initial area under the gadolinium concentration curve over 60 seconds (iAUGC_60_)	Microvasculature
Oxygen enhancing	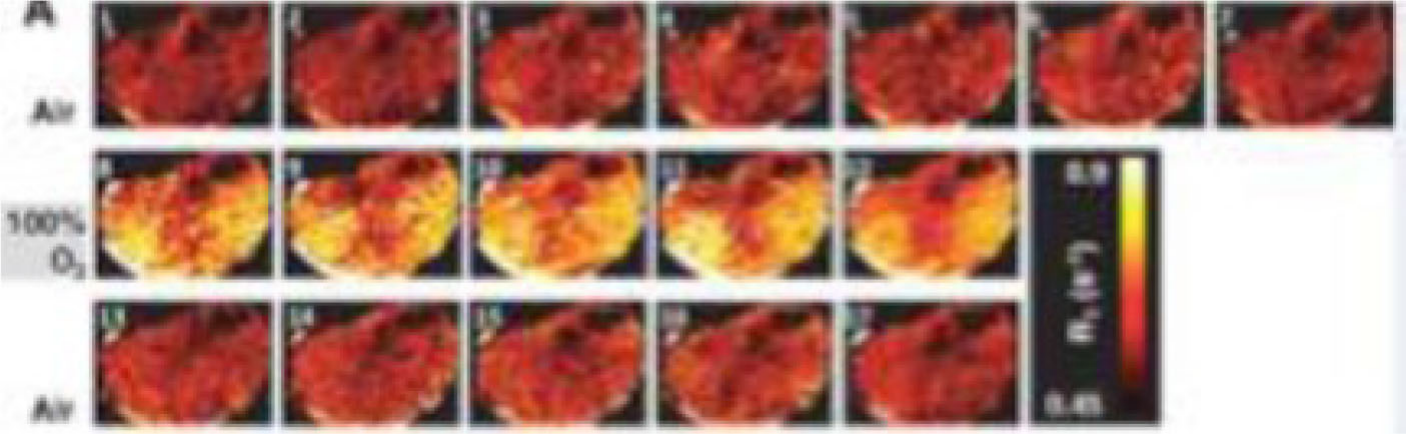	Longitudinal relaxation rate of protons (R1)	Tissue oxygenation
Magnetic resonance elastography	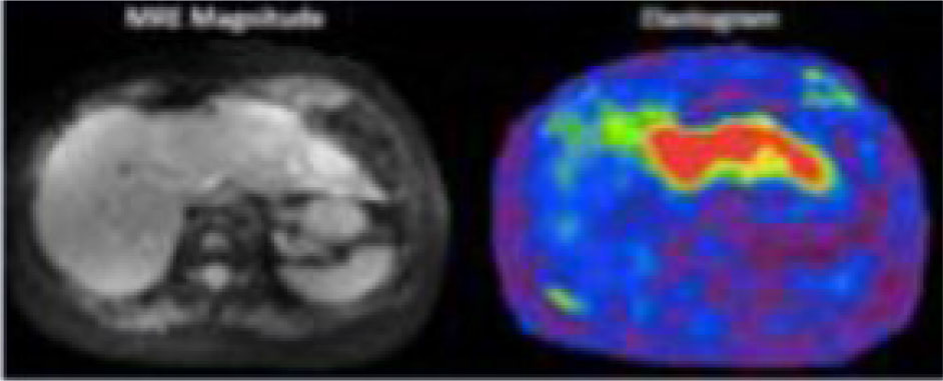	Complex shear modulus (G*)	Mechanical properties of tissue

Each imaging modality is given with an exemplar image adapted from sources listed below, an example of a parameter measurable and its proposed biological surrogate Dixon: Pre-contrast images taken of a retroperitoneal spindle cell sarcoma, showing separate in phase (water) and fat images left and right respectively which forms the basis for the calculation of fat fraction, a surrogate for fat content of tumours.

Diffusion-weighted: Example of axial diffusion weighted image on the left with corresponding ADC map on the right of the same retroperitoneal spindle cell sarcoma.

Dynamic contrast-enhanced: Example of image with overlaid K^trans^ map on the left and iAUGC_60_ on the right of a patient with a myxoid lioosarcoma of the thigh. Heterogeneity of the lesion is seen throughout the lesion signifying perfusion heterogeneity.

Oxygen enhancing: Adapted from O'Connor et al. (74). A series of R1 maps in a xenograft tumour while the mouse breathed air (top row). 100% oxygen (middle row) and back to air (bottom row) showing increase in R1 with 100% oxygen.

Magnetic resonance elastrography: Adapted from McGee et al. (23). Example of axial images of MRE magnitude on the left and masked elastogram on the right of a patient with a malignant mass in the liver, (image adapted from citation).

Schnapauff et al. in 2009 prospectively undertook DW-MRI in 30 patients prior to histological evaluation of regions of interest (ROIs), illustrating an inverse correlation of tumour cellularity and ADC, with low ADCs generated for areas of higher cellularity (77). This correlative relationship was not influenced by prior anti-cancer treatment. This finding was reproduced in two further retrospective studies, with lower mean ADC in higher grade sarcomas representative of the increased cellularity on histological analysis (78, 79). In a prospective study of 45 patients, the use of ADC to discriminate between malignant and benign soft tissue lesions was demonstrated. This study showed that malignant lesions had a significantly lower mean ADC value, further supporting the complimentary value of DW-MRI and ADC to soft tissue mass imaging (80). In addition, whilst most studies exploring ADC focus on the use of median or mean ADC values to objectively measure cellularity, this can fail to reflect baseline or post-treatment heterogeneity for any given tumour. Winfield et al. demonstrated ADC may be measured for various regions of interest on MRI, providing a deeper insight into ITH both within a region and across the entire tumour (22). An extension of DW-MRI is intravoxel incoherent motion (IVIM) which allows separation of perfusion from the true tissue diffusion. This allows calculation of quantitative parameters that reflect true tissue diffusivity (*D*), perfusion-related pseudodiffusion (D*) and perfusion fraction (*f*) which represents the contribution of water in capillaries (81). This could improve the accuracy of a quantitative parameter of diffusion by informing on the contribution of the microvascular circulation. Wu et al. demonstrated the combination of ADC and *D* were most useful in differentiating malignant and benign STS than ADC alone, and that the combination of ADC and *f* improved the differentiation between benign, intermediate and malignant STS (82). However, in Winfield et al’s study, the IVIM parameters exhibited poor repeatability when compared with ADC (22). A modification of DW-MRI is diffusion tensor imaging (DTI) and its advancement diffusion kurtosis imaging (DKI) (83, 84). These techniques add a quantitative assessment of the direction of diffusion of water molecules and may provide further diagnostic value by providing more structural information about lesions. DTI studies in muscoskeletal imaging are still in early stages but have been used to provide information about nerve fascicle visualisation for peripheral nerve sheath tumours and the integrity of axons (85–87). Similarly DKI studies remain evolutionary, however, have shown value in helping differentiate sarcoma from benign lesions and distinguishing recurrence from post surgical changes (88, 89).

Dynamic contrast-enhanced MRI (DCE-MRI) is being increasingly applied to obtain temporal information about tumour microvascularity and there have been a wealth of studies in the past few decades (90–94). It is well recognised that the tumour vascular system has an altered pathophysiology and role in tumourigenesis, supporting the diagnostic clinical application of DCE-MRI (95, 96). DCE-MRI utilises serial images capturing the arrival, duration and exit of a contrast agent in a tissue of interest, generating a signal intensity curve that may provide detailed information on both the physical and physiological properties of tissue in response to the agent (91, 97–99). The signal intensity curve and individual quantitative parameters such as the transport constant of the contrast agent from the blood plasma into the extracellular extravascular space (K^trans^), and the extracellular extravascular space volume (V_e_) may inform on underlying tissue microvasculature (99). For example tumour areas of higher perfusion and permeability having a higher and quicker uptake of contrast compared with tumours areas of necrosis (100). An early study in STS has demonstrated that DCE-MRI can aid in discriminating between low and high grade tumours, with K^trans^ differentiating grade I from grade III and grade II from grade III tumours (101). Combining K^trans^ with two semi-quantitative parameters also derived from DCE-MRI, initial area under the gadolinium concentration-time curve (iAUC) and time to peak (TTP), had the highest diagnostic performance with an area under the curve (AUC) of 0.841.

Magnetic Resonance Spectroscopy (MRS) is a technique which can provide information about the biochemical processes without the requirement for invasive intravenous contrast agents. By detecting protons (^1^H-) containing metabolites in addition to water, this can provide information about the micro-metabolic environment within tissue. In STS, a prospective study used this technique to leverage on the measurement of choline which is an established marker for increased cell membrance turnover and can indicate malignancy. The authors showed that this can be useful in differentiating grade of muscoskeletal tumours (102). In addition, in a heterogeneous lesion like STS tumours, it might highlight more aggressive areas. However, this technique has poor sensitivity and specificity in STS and can be technically challenging is some anatomical sites such as extremities (103).

### Radiomics

Recent efforts have centred on radiomic model development prediction of tumour grade given the crucial role of grade in preoperative diagnosis and treatment planning. Multiple studies have focused on identification of MRI-based radiomic features distinguishing low from high grade tumours (43, 45, 47, 78, 104). Using T2-weighted MRI images, Zhang et al. tested 5 radiomic features in a predictive model of STS grade in 35 patients with good accuracy (44). This was further explored by studies training machine learning algorithms based on radiomic features extracted from T2-weighted and/or T1-weighted MRI sequences to identify the optimal model of tumour grade prediction (43, 45). These studies used larger sample sizes (105 and 113 patients respectively) to form their complete cohort for training, testing and validation of their radiomic models, and achieved area under the curves (AUC) of over 0.9, demonstrating both feasibility and utility in their models for distinguishing grade (43, 45). In a study with the largest sample size to date, Navarro et al. retrospectively collected a training cohort of 148 patients to train a deep learning model comprising of features extracted from contrast-enhanced fat-saturated T1 and fat saturated T2 weighted images which was capable of distinguishing low from high grade tumours (105). They went on to externally validate this result in a cohort of 158 patients with AUCs of 0.75 and 0.76 for their T1 and T2 weighted radiomics models respectively. Corino et al. used DWI-MRI and it’s corresponding ADC maps in a group of 19 histologically confirmed STS patients for retrospective radiomic analysis to distinguish intermediate and high grade tumours. This study concluded that using a maximum of three features from first order statistics could achieve a model distinguishing grade with relatively good accuracy (78). Texture analysis tools can be applied to routine MR images to interrogate the inter-pixel relationships and grey level pattern of a ROI to provide a measure of heterogeneity (106, 107). This was utilised in a retrospective study of 29 patients by Meyer et al. to match Ki-67 index to discriminate between low and highly proliferating sarcomas (108). Ki-67 proliferation index is a widely used immunohistochemical test that reflects the activity of tumour cells, aiding the discrimination between low and high grade tumours, treatment response to neoadjuvant radiotherapy and can act as a predictor of clinical outcome in several cancers. Meyer et al. demonstrated several textural analyses correlated with Ki-67 index, namely run length and grey-level non uniformity (run length matrix features) had a positive correlation with Ki67 whilst sum average (grey-level histogram feature) and grey-level kurtosis (absolute gradient feature) had a negative correlation. Radiomics may be a valuable non-invasive tool for supplementing histological methods of distinguishing low and high grade STS, and may improve rates of undergrading tumours. However, MRI-based radiomic studies are usually retrospective, often limited to few imaging sequences and rarely externally validated, and therefore remain some way from constituting reliable and reproducible IBs. Despite its inferiority to MRI in soft tissue contrast, CT-based radiomics has also been explored in STS. Peeken et al. used three retrospective, independent cohorts for training, testing and validating (83, 87 and 51 patients respectively) a model to demonstrate feasibility of using CT-radiomics for grading STS, able to differentiate grade 3 from non-grade 3 tumours (47). There remains occasions, particularly for STS located within the retroperitoneum, when MRI is not conducted and therefore CT radiomics remains a useful avenue to explore. Whilst this study successfully validated its radiomics model, its AUC was 0.64, and therefore does not perform well enough to be a confident tool alongside the usual histopathological work-up for these patients.

### Image Guided Biopsy Using Next Generation Imaging

Image guided biopsy could circumvent many issues related to tissue sampling, although this has undergone relatively little change for over 30 years (109). Biopsy usually takes place under CT or ultrasound (US) guidance alone, and whilst these modalities benefit from low cost, speed, and familiarity, they do not convey the same level of biological information as next generation imaging techniques (e.g. quantitative MRI).

In other tumour types including prostate cancer bone metastases, lymphoma and neuroendocrine tumours, functional imaging has been used to select the biopsy sites thought to be most deterministic in terms of biological behaviour, response to treatment and disease outcomes (110–112). Since tumour grade is known to be an important prognostic indicator of recurrence in sarcomas, this ‘precision biopsy’ approach could be adopted to provide more accurate grading and better inform treatment decisions e.g. alternative or adjuvant therapies (113). Practically speaking, a precision MRI biopsy requires either a direct ‘in-gantry’ approach, or some form of image fusion to leverage the benefits of the two imaging modalities - either ‘cognitive’ (visual estimation) or actual (software), the latter leveraging the benefits of two imaging modalities. Sampling tumours in multiple regions would afford the ability to capture intratumoural heterogeneity and can provide an accurate ground truth for imaging biomarker validation, with a view to “virtual biopsy” in the future. For example, biopsy in a single region may not capture the necrotic or myxoid elements, which are a key component of the FNCLCC grading scheme for adult sarcomas (114). A recent example of multiregional fusion biopsy has been reported by a group who sampled different regions of ovarian tumours using CT/US fusion, guided by radiomic habitats (115). Precision biopsy would also facilitate discovery of IBs with radiogenomic study of co-localised tissue samples, integrating data-rich radiological images with biological profiling inclusive of deeper histological and molecular metrics. There is research applying “-omics” approaches such as transcriptomics and proteomics to comprehensively profile STS and their functional status, particularly in the context of heterogeneity (116–118). Further, we are increasingly understanding the importance of the role the immune environment plays within these heterogeneous tumours, and its potential to inform on behaviour and clinical outcome of STS (119–122). Combining these powerful –omics platforms with next generation imaging and biopsy techniques could accelerate the discovery and validation of IBs in STS.

## Treatment Planning and Therapeutic Monitoring

Aside from the search for IBs of histopathological phenomena, there is also a need for markers that surrogate biological features of lesions, particularly those of prognostic value that can allow better patient risk stratification. At present, the complexity of heterogeneity within STS lesions confounds our ability to individualise therapy for each patient. Upfront opportunities to identify patients at risk of relapse or poor outcome through IBs could lead to improved management strategies and clinical outcome. Furthermore, when positioned with other emerging tools such as dynamic prognostic nomograms, for example the Sarculator App (123) and PERsonalised SARComa care (PERSARC) (124), which model prognosis in STS patients using a number of clinicopathological factors, a clinical IB may improve the accuracy of these accessible and user-friendly applications for treatment planning.

Currently, radiotherapy in STS is primarily utilised in the neoadjuvant setting for tumours of the extremeties to reduce post-surgical local recurrence rates (125). This is generally preferred to radiation given in the adjuvant setting which can have long-term morbidity associated with the radiotherapy received (e.g. fractures, fibrosis and oedema) (126). A retrospective study also demonstrated that neoadjuvant radiotherapy achieved higher rates of negative surgical margins on resection of the tumour when compared with adjuvant radiotherapy and no radiotherapy (90% versus 75% and 80% respectively) (127). Unfortunately intratumoural heterogeneity causes variable treatment effect across a single tumour, wherein varying tissue composition will govern therapeutic, and up-front radiotherapy planning is difficult without improved biological information of the lesion. For example, it is known that hypoxic areas within tumours can result in chemo- and radioresistance (128, 129). Consequently a single STS tumour containing a number of hypoxic regions will fail to respond to radiotherapy in those hypoxic areas. Systemic agents may be used as neoadjuvant therapy to reduce the potential for metastasis, or in the advanced setting to reduce metastases and control symptoms (130). In high-risk STS, 50% of patients will develop advanced disease and chemotherapy forms the mainstay of management in the metastatic setting (131). The role of neoadjuvant chemotherapy is well established in certain subtypes such as rhabdomyosarcoma, however, its use in most sarcoma subtypes remains controversial (132). Available data from randomised controlled trials have significant limitations and the evidence base is insufficient (130, 133–138). In fact, the only randomised controlled trial investigating neoadjuvant chemotherapy and surgery with or without radiotherapy versus surgery with or without radiotherapy alone was not able to recruit target accrual and had a negative result (138). The addition of neoadjuvant chemotherapy was not proven to be more effective than surgery alone, with a 5-year disease free survival of 56% versus 52% respectively. Furthermore, when a histotype specific approach to neoadjuvant chemotherapy agent choice was investigated in a phase 3 multi-centre randomised controlled trial, there was no benefit observed over the standard chemotherapy regimen (133). In this study, local failure free survival at 46 months was 86% in the standard regimen group and 85% in the histotype-tailored chemotherapy group. Moreover, distant metastases free survival at 46 months was 74% for the standard chemotherapy group versus 45% for the histotype-tailored chemotherapy group. This suggests that therapeutic challenges in STS are in part related to disease rarity, however, also likely to be related to intratumoural heterogeneity. Without a greater understanding of the biology underlying this heterogeneity and the manner in which tumours may evolve in space and time, prediction of therapeutic response is difficult.

**Figure 2:**
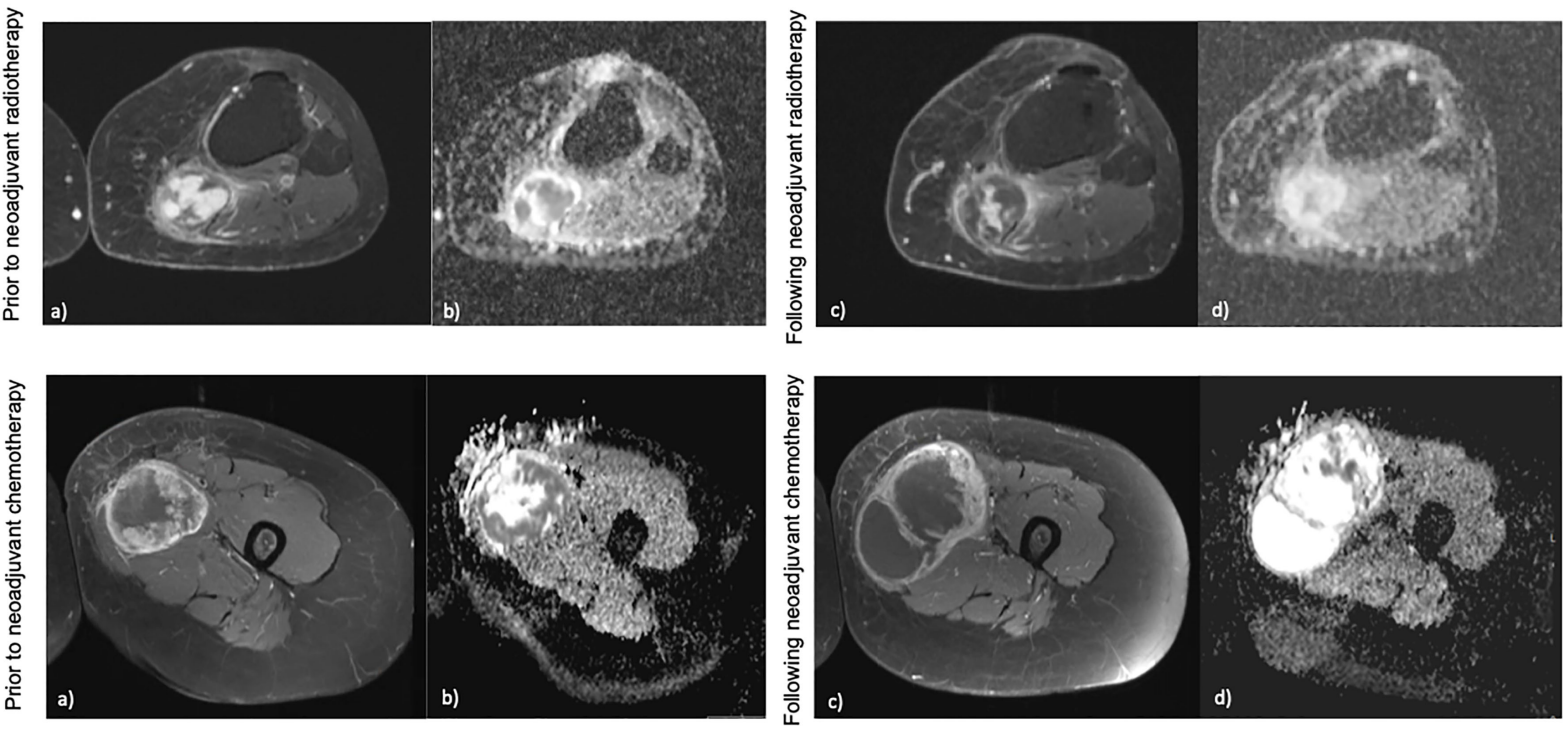
Examples of different radiological response following neoadjuvant therapy. Top: **(A, B)** demonstrate a hisloJogically confirmed myxofibrosarcoma in the calf prior to neoadjuvant radiotherapy on post-contrast imaging and ADC maps, **(C, D)** demonstrate the mass following neoadjuvant radiotherapy which shows no change in overall size, however a change in contrast signal and ADC can be seen. This would he stable by RECIST 1.1. Bottom: **(A, B)** demonstrate a biopsy confirmed ewing's sarcoma of the thigh prior to neoadjuvant chemotherapy on post-contrast imaging and ADC maps, **(C, D)** demonstrate the mass following neoadjuvant chemotherapy which has increased in size, however a change in ihe contrast signal and ADC within the mass can be seen. This mass would be measured as progression by RECIST 1.1 criteria.

Furthermore, whilst serial biopsies theoretically serve to monitor therapy, this requires multiple invasive and sometimes technically challenging procedures, which remain prone to sampling error (125). In contrast, imaging affords non-invasive, global, and temporal assessment of tumours. However, radiological response to treatment is not reliably characterised with conventional size-based measurements on imaging in STS. At present, Response Evaluation Criteria for Solid Tumours (RECIST 1.1) is used to measure radiological response to therapy based on whether a tumour demonstrates shrinkage, an increase in size or no change in overall size on imaging scans (139, 140). In the neodjuvant setting there is a disparity between the therapy response measured radiologically and the histopathological response findings on tissue (141–143). Treatment induced necrosis on tissue examination is an independent marker of prognosis, and dimension-based imaging is a crude predictor of this (144, 145). Numerous radiological patterns of response can be seen and good responders following neoadjuvant therapy as defined by RECIST 1.1 may be rare (**Figure 2**) (141). A significant reduction in the volume of responding tumours following radiotherapy has been reported as low as 0%, and instead tumours may grow due to cystic transformation, haemorrhage and necrosis (pseudoprogression) (141, 142, 146, 147). In the phase 3 randomised controlled trial investigating the histotype approach to neoadjuvant chemotherapy, no patients achieved complete response and only 14% achieved a partial response with the rest being objectively measured as stable or progressive disease (133). A retrospective review conducted on patients with advanced sarcoma demonstrated that good responders (27% of the cohort defined as complete and partial responders) following neoadjuvant chemotherapy had comparable rates of relapse and overall survival to the non responders (148). A study by Delaney et al. demonstrated that in a prospectively recruited group of 48 STS patients treated with neoadjuvant chemotherapy and radiotherapy prior to surgery, 6 patients had progression as measured by RECIST 1.1 (149). However, on histological examination of the surgical specimen, 4 of the 6 patients had prominent necrosis (more than 90%) of the sample confirming good sensitivity to the neoadjuvant therapy received. Further, a previous study observed 31% of STS tumours increasing in size by more than 10%, with no association with a deterioration in clinical outcome (150). Efforts to establish alternative response criteria have been undertaken. The Choi criteria measures radiological response to therapy based on changes in tumour size and/or density (tumour attenuation measured in Hounsfield units (HU)) on computed tomography (CT) (151). Studies have demonstrated that density changes often preceed any dimensional response to the tyrosine kinase inhibitor imatinib in gastrointestinal stromal tumour, and therefore Choi criteria have a higher sensitivity to tyrosine kinase inhibitor benefit in these patients (151, 152). In other STS subtypes, Choi criteria have shown promise as superior predictors of histopathological response to cytotoxic chemotherapy, and a modified Choi criteria can allow assessment on MRI scans (153, 154). However, these criteria require appropriate software for postprocessing and the segmentations required impact on radiologist time. This has hindered validation, including reproducibility in larger prospective studies.

To overcome the limitations of current therapy response criteria, the capacity to gain serial virtual biopsies by next generation imaging received as part of a patient’s pathway could improve treatment monitoring. If a robust IB can be developed, alternative and more precise monitoring of therapy is possible. Not only could this allow earlier alterations in therapeutic regimes for patients who are not responding, it would also improve dosing options and reduce the likelihood of severe adverse side effects. This IB may be used independently or again in concurrence with other therapy monitoring methods such as the use of tumour circulating-derived DNA(ctDNA) and tumour cell free DNA(ftDNA) (155) as markers of response as they translate into clinical practice. This in turn would bring us closer to the goal of personalised medicine and allow clinical decisions to be based of individualised tumour and patient characteristics that are achievable in a non-invasive and dynamic way.

### PET/CT and PET/MRI

Studies involving ^18^F-FDG PET/CT have shown value in using the reduction in SUVmax following treatment to indicate possible therapeutic response even when overall tumour volume has not reduced (74, 156–158). Benz et al. demonstrated that a decrease in SUVmax of ≥ 35% from baseline to post-treatment scans was a sensitive predictor of histopathological response to neoadjuvant therapy in a group of 50 patients with resectable high grade STS (156). Schuetze et al. demonstrated a reduction in SUVmax of ≥ 40% was an independent predictor of improved prognosis (disease specific and overall survival) in 46 patients with high grade sarcoma receiving neoadjuvant chemotherapy (157). However, these PET/CT studies are limited to small patient numbers, single centres and are usually retrospective and therefore, confidence in SUVmax as a clinical IB will be dependent on ongoing prospective studies.

PET/MRI has also shown promise for monitoring of patients, adding the excellent soft tissue contrast possible with MRI to information obtained through PET. A study by Erfanian et al. reported improved accuracy in the detection of local recurrence of STS of 90% when compared to conventional MRI alone at 83% (159). Like PET/CT, progress for this imaging modality will rely on robust prospective studies. A further exciting avenue for PET research in STS is immuno-PET imaging as a potential non invasive and serial tool to monitor pre-selected immune markers or aid the selection of patients for immunotherapy. This remains in early stages, however, a study demonstrated feasibility of using a radiotracer and immuno-PET to detect and monitor programmed cell death protein 1 (PD1, an immune checkpoint protein) in humanised mouse models (160).

### Quantitative MRI

Frequently ADC values change following radiotherapy signifying possible treatment response, even when overall tumour volume remains unchanged (22, 161–163). Winfield et al. also illustrated that in the same group of retroperitoneal STS, patient MRIs following radiotherapy demonstrated an increase in ADC, perhaps reflecting biological changes to a less cellular tumour suggestive of response. In this study, ADC was shown to have excellent repeatability, with a coefficient of variation (CoV) of 2.5% for median ADC (22). The repeatability of ADC adds to the confidence of its usage as a robust, standardised IB. However, it is important to note that at present, there remains no standardised method to measure ADC in STS, particularly with relation to therapeutic response (164). This can range from variations in the acquisition of diffusion-weighted images, the ROI selection process (for example 2D ROIs versus 3D volume ROIs) and the ADC parameter selected (for example minimum ADC versus mean ADC) (22, 77, 162). As such, this hinders our ability to standardise study findings and like other IBs, it also suffers from lack of reproducibility and validation efforts in prospectively designed studies.

DCE-MRI has also been used to demonstrate changes following therapy, suggestive of possible response (98, 165–168). Recently, in a subset of 10 patients with myxoid liposarcoma from the Dose Reduction in Preoperative Radiotherapy in Myxoid Liposarcomas (DOREMY) multicentre clinical trial, a higher baseline K^trans^ was linked to response following neoadjuvant radiotherapy and may allow prediction of early response to radiotherapy (169). Although time consuming analysis and some limitations on anatomical coverage preclude routine clinical use, DCE-MRI represents an interesting avenue for further research into the STS vascular microenvironment and its role in therapeutic response.

Oxygen enhanced (OE-)MRI is an emerging technique that may offer insight into tumour hypoxia; a well-recognised negative prognostic factor in cancer (170–172). To date, there remains an unmet need to measure hypoxia through a biomarker, and MRI offers an attractive non-invasive and serial option (173). OE-MRI is capable of mapping and quantifying the spatial distribution of tumour oxygen delivery. By measuring the longitudinal relaxation rate of hydrogen nuclei (R_1_), which changes in relation to the dissolved oxygen concentration in tissue or plasma, OE-MRI can characterise well- and poorly-oxygenated tissue. Inhalation of hyperoxic gas results in delivery of excess oxygen to well-oxygenated tissue, however, given well- oxygenated tissue will have saturation of haemoglobin molecules with oxygen, the excess oxygen remains within the plasma and interstitial tissue fluid, increasing the tissue R_1_. Preclinical and clinical studies in renal cell carcinoma, glioma, prostate cancer and lung cancer have demonstrated the feasibility and accuracy of OE-MRI in mapping hypoxic subregions, including changes following radiotherapy (173–179). Whilst human studies in sarcoma are lacking, the early work by Cao-Pham et al. in rhabdomyosarcoma xenograft models showed that R_1_ as measured by OE-MRI is sensitive to changes in oxygenation (180). Further exploration in STS is required, however in a group of heterogeneous cancers, OE-MRI could optimise our treatment planning for these patients. Visualising areas of hypoxia across a tumour could aid radiotherapy planning, stratifying patients accordingly.

Similarly, magnetic resonance elastography (MRE) is gaining attention as a quantitative imaging method capable of assessing the mechanical properties of tissue. MRE informs on the viscoelastic properties of tissue by measuring the propagation of mechanical waves through tissue (181). Already a well-established technique in patients with chronic liver disease, there is a strong motivation to explore the applicability of MRE to other pathologies (182, 183). Preclinical studies have demonstrated that altered tissue “stiffness” (quantified using the complex shear modulus (G*) and related parameters) can be used to indicate malignant masses within brain parenchyma, and that changes in mechanical properties of a tumour may precede volume change following treatment indicative of response in colon cancer and non-Hodgkin’s lymphoma (181, 184–186). There are currently limited studies within STS, however, Pepin et al. demonstrated technical feasibility of MRE in a pilot study of 13 sarcoma patients, and possible utility in assessment of early response to radiation therapy (in 4 of these 13 patients) (187). Further studies are underway, and this may offer a further non-invasive method to interrogate deep and complex masses such as those within the retroperitoneum.

### Radiomics

Much of the radiomic research in STS is focused on predicting therapeutic response and clinical outcome. In an externally validated study, Spraker et al. found that features extracted from T1-weighted MRI were independently associated with overall survival (OS), and a radiomics model combined with clinical features (age and grade) performed best (46). In a further study, the addition of T2-weighted MR radiomics to the clinical American Joint Committee on Cancer (AJCC) staging system into a nomogram improved the clinical net benefit for stratification of patients for OS, and was superior to using AJCC system alone (188). The same group went on to apply deep learning to the two cohorts (that had been slightly expanded), concluding that the addition of deep learning to their previous radiomic model yielded comparable prediction performance for OS. This finding was supported by a further nomogram constructed by Yan et al, demonstrating successful patient risk stratification based on progression free survival (PFS) when MR-based radiomics was used in addition to clinical factors, with an increased benefit to using AJCC staging system alone (189). In CT, the same study by Peeken et al. using radiomics to distinguish tumour grade in STS explored the predictive performance of radiomic features for OS, distant progression free survival (DPFS) and local progression free survival (LPFS). This study concluded that again, despite CT’s inferior soft tissue contrast, there was value in using a radiomics model to stratify patient risk based on their survival outcomes. This finding was successfully validated in an external cohort, and results were more consistent than using a clinical model (47).

In the context of response to therapy, delta-radiomics, the change in a given radiomics feature in a set of longitudinal images is of particular interest. Gao et al. used longitudinal DW-MRI images in 30 STS patients receiving neoadjuvant RT at three time points, to develop a predictive model of pathological therapeutic response (190). The model demonstrated that radiomic features alone were not sufficient to predict response, however, the inclusion of delta-radiomics features at mid- and post-therapy time points optimised prediction of response to RT. Delta-radiomics was also used for prediction of pathological response to neoadjuvant chemotherapy in a retrospective cohort of 65 STS patients. A radiomics model was developed based on longitudinal MRI scans taken at baseline and following two cycles of anthracycline-based therapy, prior to surgical excision from 50 of the 65 patients. The findings were then validated in the remaining 15 patients. Using 3 STS features of shape, heterogeneity and surrounding tissue, the study concluded an improved performance of the radiomics model when compared to RECIST 1.1, and a promising tool for the evaluation of early response following only two cycles of chemotherapy (50).

### Multispectral Tissue Characterisation and Habitat Mapping

**Figure 3:**
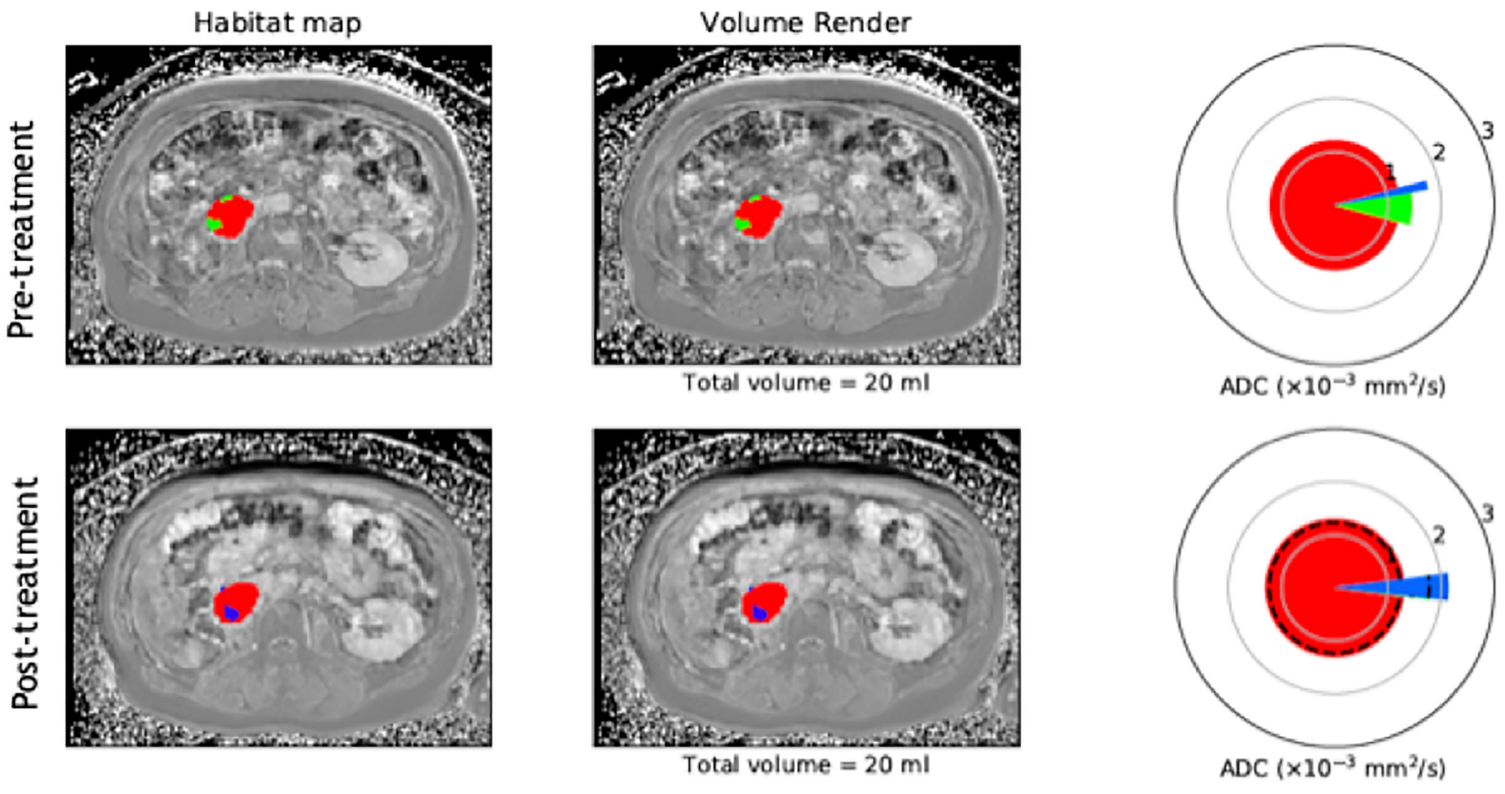
Example of habitat imaging as demonstrated by a supplementary figure provided by Blackedge et al. (21). Axial MRI scan taken pre and post-radiotherapy in a patient with a retropentoneal STS tumour. Habitat maps are overlaid on MRI scans acquired for this patient and volume renders shown. The different colours within the mass on MRI scans represent the different sub compartments assigned according to the qMRI parameters EF. FF and ADC. Tissue sub compartments with high ADC and low FF are represented in blue and may suggest necrotic or cystic tissue, low ADC and high EF in red and suggest cellular vascular tissue, and low ADC and low EF in green and suggest poorly vasculansed tissue. Spie charts show the average ADC for each tissue sub compartment as the illustrated radius of each segment, whilst the angular proportion represents the proportional volume of habitat class. Pre and post-treatment, the colours within the mass are shown to change, with loss of the green sub compartment (poorly vasculansed tissue) and increase in the blue sub compartment (necrotic/cystic tissue), signifying a change in tissue characteristics following therapy and possible response even when overall volume is unchanged. (Figure adapted from citation).

Previous studies have utilised quantitative parameters derived from diffusion weighted imaging, namely ADC, the transverse relaxation time (T_2_) and proton density (M_0_) to objectively segment tumours into distinct subregions and in relation to therapy (191–195). In a xenograft mouse model of radiation-induced fibrosarcoma (RIF-1), Henning et al. was able to segment tumours into regions based on two viable tumour groups and two non viable (necrotic) tissue groups which were confirmed on histopathological staining of a hypoxia marker (192). The group also analysed the viable tissue groups further and characterised them into well oxygenated radiosensitive and hypoxia and radioresistant groups (193). This is important ground work for methods of objectively characterising tissue based on quantitative markers for underlying biological properties. Building on this are habitat maps which incorporate multiple quantitative parameters obtained from numerous MRI sequences. These maps characterise tissue into distinct biological habitats based on their combination of multiparametric MRI parameters and provide insight into multiple aspects of biological heterogeneity and their interaction with one another (21). In a cohort of 30 retroperitoneal STS, automated segmentation of tumours into tissue subtypes based on fat fraction (FF), enhancement fraction (EF) and ADC, demonstrated distinct habitat maps and changes following radiotherapy suggestive of response, including when overall tumour volume remained the same or increased (**Figure 3**). These techniques may improve our understanding of the differing characteristics within this heterogeneous cancer and how distinct regions respond variably to the same treatment. In addition, it can illustrate results in a timely and clinician friendly manner that is not reliant on interpretation of numerical values, easing its transition into practice.

### Ongoing Challenges and Future Directions

The integration of powerful imaging technology to inform on the biology of these heterogeneous tumours is needed in the clinical setting for this complex disease. Indeed, there is potential to enable a more personalised approach to patient management and uncover candidate therapeutic options. However, there are a number of challenges that need to be addressed prior to the successful implementation of “virtual biopsy” methods into practice. If the ultimate goal of quantitative and radiomic imaging techniques is the development of an IB, consideration of the steps of biomarker development is required (5). At present, despite a heavy investment into the search for IB discovery, most sarcoma work has faltered at the initial stage of technical (assay) validation (22).

### Reproducibility and Complexiy of Data

Quantitative MRI sequences have a varying complexity of delivery, particularly those which require intricate post-processing of images, and therefore, make reliable and confident translation across radiology departments challenging. This is mirrored in the variation seen across different MRI scanners in image acquisition and measurement output, usually requiring adaptations at every centre and vendor. This weakens the reproducibility potential of quantitative MRI techniques and may oblige different centres to perform reproducibility/repeatability studies to conclude some clinical capability. As a result, most studies remain limited to a single centre using the same MRI machine. This hinders the progression of novel and promising quantitative MRI techniques through imaging biomarker validation. Similarly, a crucial step in the radiomics workflow is standardisation of acquired images to allow reliable image delineation and radiomic feature extraction, and this can pose significant challenges at multi-centre level. Each step in the radiomics workflow can in fact affect results, and similar to quantitative MRI, requires careful or expert processing of data. This makes reproducibility efforts challenging. To date, few studies have spanned multiple centres, and the majority of studies have not been tested in an independent external cohort. Attempts to provide standardised guidance for the translation of acquired imaging to high-throughput IBs are underway. The Image Biomarkers Standardization Initiative (IBSI), an independent international collaboration aims to provide detailed guidance on the mandatory steps for radiomic analyses, and Lambin et al. have defined the Radiomics Quality Score (RQS) to provide an objective estimate of the quality of the study (196, 197). Future studies may benefit from these to aid transition from proof of concept to real life clinically applicable tools.

### Limited Cohort Size and External Validation

STS is a disease of rarity, and it’s diverse histological subtype profile confounds effort to accrue patient cohorts comparable to other cancer types. This can make assessment of the quality of findings within imaging studies challenging. In particular, radiomics is dependent on large datasets for reliable feature extraction and robust conclusions to be drawn from results. Furthermore, a crucial component of translating an IB is it’s independent validation in an external cohort, which is a recognised challenge in the sarcoma community. Whilst there are methods to deal with smaller datasets such as cross-validation, these still remain limited to the individuals studied and findings cannot be generalised to the rest of the population, not overcoming the issues pertaining to external validation (198).

A solution to this may be data-sharing. A powerful drive through the NIH’s Quantitative Imaging Network (QIN) and its leveraging on The Cancer Imaging Archive (TCIA) has demonstrated feasibility in data sharing of clinical images across various sites (199). This partnership holds promise to overcome challenges related to data-sharing, facilitating multi-site collaboration and support the development of IBs through validation (200–202). Builiding on this, there are efforts to harmonise quantitative MRI data across centres and develop a robust infrastructure to allow this, such as the National Cancer Imaging Translational Accelerator (NCITA) consortium which includes 9 UK centres aiming to develop a robust multicentre IB pipeline (65). This would provide opportunities for both multi-centre data to increase the size of initial datasets and external cohorts for the validation of discoverable IBs.

### Retrospective Study Design

The challenges related to the low incidence and heterogeneity of the disease can in turn make prospective collection of data difficult. It can take multiple years to recruit a desired cohort size, and studies may be closed early upon failing to reach target accrual. As such, alongside the readily available retrospective data, many imaging studies within STS remain retrospective in nature. This carries risk of confounding and bias (such as selection or recall bias), and results in an inferior level of evidence. Furthermore, retrospective designs negate the ability to carry out repeatability tests for quantitative MRI and radiomic studies due to a lack of repeat scans performed around the time of the initial scan. Going forward, confident validation and translation of IBs is heavily dependent on prospective studies.

Within STS, a Cancer Research United Kingdom (CRUK) Sarcoma Accelerator consortium partnering centres across the UK, Spain and Italy aims to develop a multicentre, expansive data pipeline to develop clinical and biological models to further our understanding of sarcoma biology in patients with high risk STS undergoing perioperative management. This will involve prospective collection of imaging data across 5 years and spanning these multiple centres. This will greatly benefit IB discovery and validation in sarcoma, where limited patient numbers for studies will increasingly rely on close multi-centre collaborations and data-sharing to propel their progress to clinical translation.

## Conclusion

**Figure 4:**
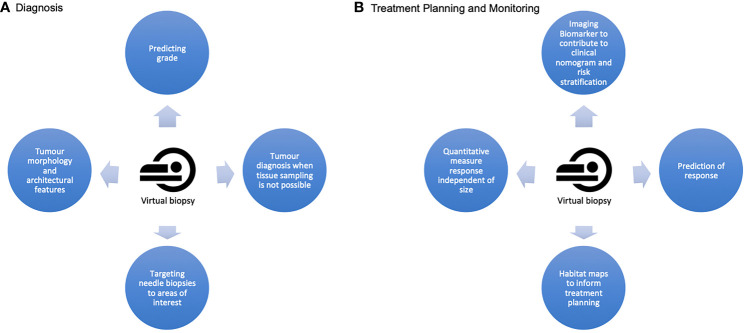
A summary of the potential applications of a virtual biopsy at the two crucial stages of a sarcoma patient's cane pathway **(A)** At diagnosis, a virtual biopsy can offer quantitative imaging biomarkers to improve accuracy of grading, complimentary architectural information about the lesion and information about diagnosis and grade when needle biopsy is not possible. It can also guide precision needle biopsy. **(B)** For treatment planning and monitoring, virtual biopsy offers the opportunities for improved patient risk stratification and prediction of therapeutic response. It can provide information assimilated in habitat maps to capture global and spatial heterogeneity for improved treatment planning, and provides the potential for serial quantitative, non invasive measures of response which are independent of tumour size.

The ambition to achieve non-invasive and global captures of tumour biology remains a priority in complex and heterogeneous malignancies such as STS. Whilst radiomics is promising, sub-optimal datasets and a lack of external validation mean this is some way from becoming a tool to direct patient care. However, we can now offer virtual biopsies in the research setting following the validation of a number of IBs in sarcomas, and which can be assimilated as habitat maps (**Figure 4**). The clinical impact of identifying these distinct imaging phenotypes is yet to be fully explored and as further IBs including radiomic signatures mature, they will also be incorporated into these models of heterogeneity. The next step is prospective clinical trials exploring habitat map-guided risk stratification, response assessment and adaptive therapies on patient outcomes. Translation to routine clinical care will also be ultimately reliant on improved proficiencies within radiology departments to seamlessly incorporate the necessary post-processing and data visualisation tools into routine workflow.

## Author Contributions

AA, EJ, JW, RJ, PH, and CM: literature search. AA, JW, MB, CM, and PH: figures. All authors contributed to manuscript revision and approved the final version.

## Funding

This report is independent research funded by the National Institute for Health Research.

## Conflict of Interest

RJ is the recipient of grants/research support from Merck Sharp & Dohme and GlaxoSmithKline. RJ is the recipient of consultation fees from Adaptimmune, Athenex, Blueprint Medicines, Clinigen, Eisai, Epizyme, Daichii, Deciphera, Immune Design, Lilly, Merck, PharmaMar and UpToDate.

The remaining authors declare that the research was conducted in the absence of any commercial or financial relationships that could be construed as a potential conflict of interest.

## Publisher’s Note

All claims expressed in this article are solely those of the authors and do not necessarily represent those of their affiliated organizations, or those of the publisher, the editors and the reviewers. Any product that may be evaluated in this article, or claim that may be made by its manufacturer, is not guaranteed or endorsed by the publisher.
